# Clarifying the Diagnostic Criteria for Lemierre Syndrome

**DOI:** 10.1002/ccr3.72573

**Published:** 2026-04-17

**Authors:** Steven H. Yale, Halil Tekiner, Eileen S. Yale

**Affiliations:** ^1^ Department of Medicine University of Central Florida College of Medicine Orlando Florida USA; ^2^ Department of the History of Medicine and Ethics Erciyes University School of Medicine Kayseri Türkiye; ^3^ Nova Southeastern University Medical School, Kiran C. Patel College of Allopathic Medicine Fort Lauderdale Florida USA

**Keywords:** anaerobic bacteria, diagnosis, Lemierre syndrome, odontogenic infections, septic pulmonary embolism, thrombophlebitis

## Abstract

This letter clarifies the case definition of Lemierre syndrome and emphasizes that this case represents an otogenic infection with internal jugular vein thrombosis. Accurate diagnostic terminology guides antimicrobial selection, defines prognosis, ensures comparability across future case series and systematic reviews, and aids physicians in recognizing this often overlooked “forgotten disease.”

To the Editor,

1

The case reported by Abdela et al., “Unmasking rarity: An atypical presentation of Lemierre's Syndrome,” raises important diagnostic considerations regarding the definition of Lemierre syndrome and whether the reported patient met these criteria [1].

In his 1936 address to the Middlesex Hospital Medical School, André Lemierre described a syndrome characterized by high fever, tonsillitis, and septic pulmonary emboli. Additional findings included submandibular gland enlargement, lateral neck pain, and edema extending from the mandible to the clavicle along the sternocleidomastoid muscle [2]. He emphasized that septic thrombophlebitis could begin in the tonsillar or peritonsillar veins and extend to the facial or internal jugular veins, a continuum previously recognized by Eugen Fränkel [2, 3].

The patient described by Abdela et al. had an otogenic infection rather than a tonsillar or peritonsillar source [1]. Although the clinical picture included internal jugular vein thrombosis, the infection's origin differs from that in Lemierre's original description. Other observations have been reported by Cloutet et al. and George et al., who documented thrombosis of the facial or presumed peritonsillar veins without internal jugular involvement, yet with a pharyngeal infectious focus [4, 5]. Therefore, thrombotic involvement of the internal jugular vein is not an absolute prerequisite for Lemierre syndrome, but a pharyngeal, peritonsillar, or facial source, high fever, tonsillar or peritonsillar involvement and septic pulmonary emboli are fundamental.

Anaerobic organisms that are native to the oropharynx are usually linked to Lemierre's condition from a microbiologic and pathophysiologic perspective, reflecting its unique anatomic origin. Abdela et al. case lacks a pharyngeal focus, suggesting a distinct pathophysiology more in line with otogenic infection than typical Lemierre syndrome.

Based on Abdela's review of the literature (2008–2022), they identified 11 cases of otogenic infections and Lemierre syndrome, two of whom had septic pulmonary emboli and presumed jugular‐vein thrombophlebitis, but such cases represent otogenic acute, suppurative or chronic otitis media and, in some cases, sepsis or septic thrombophlebitis rather than variants of Lemierre disease [6]. Accurate diagnostic terminology is not merely semantic. It guides antimicrobial selection, defines prognosis, and ensures comparability across future case series and systematic reviews. Furthermore, it may help physicians in recalling this rare and unfamiliar “forgotten disease.” Accordingly, the case presented by Abdela et al. would be more accurately described as a complicated otogenic infection with internal jugular vein thrombosis rather than Lemierre‐like syndrome or any atypical presentation of Lemierre disease.

## Author Contributions

**Steven H. Yale:** conceptualization, writing – original draft, writing – review and editing. **Halil Tekiner:** conceptualization, writing – original draft, writing – review and editing. **Eileen S. Yale:** conceptualization, writing – original draft, writing – review and editing.

## Funding

The authors have nothing to report.

## Ethics Statement

The authors have nothing to report.

## Consent

The authors have nothing to report.

## Data Availability

The authors have nothing to report.

## References

[1] H. A. Abdela , T. G. Woyimo , K. N. Muktar , et al., “Unmasking Rarity: An Atypical Presentation of Lemierre's Syndrome,” Clinical Case Reports 13, no. 9 (2025): e70844, 10.1002/ccr3.70844. 40919388 PMC12408373

[2] A. Lemierre , “On Certain Septicaemias due to Anaerobic Organisms,” Lancet 227 (1936): 701–703, 10.1016/S0140-6736(00)57035-4.

[3] E. Fränkel , “Über postanginöse Pyämie [On Postanginal Pyemia],” Virchows Archiv: Pathological Anatomy and Physiology and Clinical Medicine 254 (1925): 639–655, 10.1007/BF01909789.

[4] A. Cloutet , R. K. Botta , S. R. Kulkarni , and P. K. Ponna , “A Complex Case of Lemierre's Syndrome With Facial Vein Involvement,” Cureus 14 (2022): e23420, 10.7759/cureus.23420. 35475072 PMC9028854

[5] A. George , J. Thomas , S. Welikumbura , and Y. Achhapalia , “Lemierre's Syndrome: An Atypical Case of *Fusobacterium necrophorum* Bacteraemia in the Absence of Internal Jugular Vein Thrombosis,” Cureus 16 (2024): e66867, 10.7759/cureus.66867. 39280464 PMC11397421

[6] C. S. Mattar , R. L. Keith , R. P. Byrd, Jr. , and T. M. Roy , “Septic Pulmonary Emboli due to Periodontal Disease,” Respiratory Medicine 100 (2006): 1470–1474, 10.1016/j.rmed.2005.11.010. 16376534

